# Female reproductive tract infections: understandings and care seeking behaviour among women of reproductive age in Lagos, Nigeria

**DOI:** 10.1186/1472-6874-10-8

**Published:** 2010-03-23

**Authors:** Kabiru A Rabiu, Adeniyi A Adewunmi, Fatimat M Akinlusi, Oluwarotimi I Akinola

**Affiliations:** 1 Department of Obstetrics and Gynaecology, Lagos State University Teaching Hospital, Ikeja, Lagos State, Nigeria

## Abstract

**Background:**

Reproductive tract infections (RTI's) are endemic in developing countries and entail a heavy toll on women. If untreated, RTI's can lead to adverse health outcomes such as infertility, ectopic pregnancy and increased vulnerability to transmission of the human immunodeficiency virus. It is also associated with adverse pregnancy outcomes. While RTI's and its sequelae abound in Nigeria, there is paucity of publications on the subject in the country. This study assessed the understandings and care seeking behavior with regards to RTI's among women of reproductive age in Lagos, Nigeria with the aim of improving awareness on the subject.

**Methods:**

A descriptive cross sectional survey of women attending the gynaecological outpatient and family planning clinics of the Lagos State University Teaching Hospital was carried out between 1^st ^June 2008 and 31^st ^August 2008 using a pre-tested questionnaire. Data was analysed using the Epi-Info 3.5 statistical software of the Centre for Disease Control and Prevention, Atlanta U.S.A.

**Results:**

Most of the respondents (77.2%) had heard of RTI's. Toilet was the most perceived mode of contracting RTI's (44.6%), followed by sexual intercourse and poor hygiene. Vaginal discharge was the commonest symptom of RTI's named while inability to get pregnant was the commonest named complication. Majority of the respondent's demonstrated poor overall knowledge of symptoms and complications of RTI"s. 37.4% of the respondents had experienced symptoms of RTI's in the preceding six months. Vaginal discharge was the commonest symptom reported (21.8%) and the majority of those who reported symptoms sought medical treatment. Government health centres were the most visited health facilities for treatment.

**Conclusion:**

Even though most of the respondents have heard of RTI's and sought treatment when symptomatic, they demonstrated poor overall understanding of the subject. There is need to educate women on preventive strategies, as RTI's are often assymptomatic.

## Background

The global burden of reproductive tract infections (RTI's) is enormous and of a major public health concern, particularly in developing countries where RTIs are endemic [1].

RTI's, excluding Human Immunodeficiency Virus (HIV) constitute the second major cause of disease burden (after maternity related causes) in young adult women in developing countries[1].

RTI's cover three types of infections [2,3]: Sexually transmitted infections (STI's); infections that result from overgrowth of organisms normally present in the reproductive tract and infections associated with medical procedures including abortion and insertion of intra uterine devices. Female RTI's usually originate in the lower genital tract as vaginitis or cervicitis and may produce symptoms such as abnormal vaginal discharge, genital pain, itching and burning feeling with urination. However, a high prevalence of asymptomatic disease occur which is a barrier to effective control [4]. Even when symptoms occur, their presentation can overlap with and be diagnosed as a normal physiological change and normal physiological discharge may be misdiagnosed as RTI's [5].

Also the absence of approachable and comprehensive reproductive health services often means these symptoms must be tolerated stoically as an unfortunate but familiar reality of reproductive life [4].

In some instances, despite availability of these services, symptomatic persons do not seek or delay seeking appropriate diagnostic and treatment services [6].

RTI's entail a heavy toll on women; if untreated can cause serious consequences of infertility, ectopic pregnancy, cervical cancer, menstrual disturbances, pregnancy wastage and low birth weight babies [1]. The presence of RTI's especially ulcer causing STI's can enhance the acquisition and transmission of the human immunodeficiency virus [7].

Infertility is a major health problem in Africa, particularly in sub-Saharan Africa where 20-30% of couples are unable to conceive [8]. Most health advocates regard infertility as the most important reproductive health and social issue confronting Nigerian women and gynaecologists frequently report that infertility constitutes 60%-70% of their consultations in tertiary institutions [9]. In Nigeria, most cases of infertility follow RTI's [10].

Ectopic pregnancy constitutes a large percentage of acute gynaecological emergencies in Nigeria and is an important cause of maternal mortality [11-13]. A study in Lagos, Nigeria found previous STI's and pelvic inflammatory disease as major risk factors for ectopic gestation [14].

Cervical cancer is usually the result of sexually transmitted infection and human papilloma virus is the causative agent. It is the commonest reproductive tract malignancy and a leading cause of cancer death in Nigerian women [15]. In contrast to most other cancers, it is common below the age of 50 years and is therefore an important cause of premature death [16]. Other sequelae of RTI's also have enormous impact on the population [2,3].

Despite its enormous implications for women's health, the degree to which RTI's have been neglected by both the local and international health community is alarming. In allocating scare human and financial resources, most policy makers, programme planners and donor agencies consistently give low priority to control of these infections.

The current HIV/AIDS pandemic has undoubtedly done more havoc to shift attention from other RTI's. At this time of worldwide financial crisis, information on reproductive morbidities is essential in ensuring appropriate allocation of scarce resources and planning of cost effective health care strategies. To initiate any meaningful preventive measures for control of RTI's, there is need to create awareness and improve knowledge on the subject.

This study was carried out to assess the understandings and care seeking behavior with regards to RTI's among women of the reproductive age. Those patients attending the gynaecological and family planning outpatient clinics of the Lagos State University Teaching Hospital form a readily available and accessible group.

## Methods

### Design

The study was a descriptive cross sectional survey.

### Setting

The study was conducted at the gynaecological out patient and family planning clinics of the Lagos state university teaching hospital (LASUTH), which is one of the two main referral hospitals with obstetrics and gynaecological services in metropolitan Lagos.

The gynaecological outpatient clinic takes place between 09.00 and 16.00 hours every working day of the week. The patients seen are referred from private hospitals, general hospitals within and outside Lagos as well as from other departments of LASUTH. An average of about 60 patients attend the clinics each day and about 20% of these are new patients

The family planning is open between 08.00 and 16.00 hours (Monday to Friday). Patients either walk into the clinic or are referred from the gynaecological or post natal clinics. An average of 20 patients attends the clinic each day.

### Study Population

The participants in this study were the new patients who attended the gynaecological and family planning out-patient clinics of LASUTH between 1^st ^June and 31^st ^August, 2008. A total of 500 questionnaires were administered. Although the response rate was 100%, 13 respondents were excluded because of observed inconsistencies in response to questions. Data was therefore complete in 487 respondents.

### Data Collection

The instrument for data collection was a pre-tested questionnaire. The purpose of the study was explained to the patients and those who consented were interviewed by the authors, and medical students who had been carefully briefed about the technique of administration. Confidentiality was maintained by not including their names and addresses so as to elicit correct responses.

The technique of face to face interview was used for data collection.

The questionnaire was in three parts;

The first part solicited for socio-demographic characteristics of the respondents; the second part assessed their understandings of RTI's, while the third part assessed care seeking behaviour. We specifically asked them if they knew whether any of the four commonly chosen contraceptive methods in our centre (Condoms, intrauterine contraceptive devices, oral contraceptive pills and implants) increase, decrease, or does not have any effect on the transmission of RTI's. All the questions were constructed in the same style and were either direct (single or multiple) or open ended.

### Analysis

The open ended questions were first categorized and all the data obtained were entered into the computer and analysed using EPI - info 3.5 statistical software (2008 version) of the Centre for Disease Control and Prevention Atlanta, USA and presented in descriptive and tabular forms as frequencies and percentages.

### Ethics

Formal approval for the study was obtained from the Research Ethics Committee of the Lagos State University Teaching Hospital. Participation of the patients was voluntary and only those who gave their consent after the purpose of the study had been explained to them participated in the study.

## Results

A total of 500 new patients from the gynaecological and family planning out patients clinics of the Lagos State University Teaching Hospital who gave their consent participated in the study. Data were complete for analysis in 487(97.4%) Table 1 summarises the socio-demographic characteristics of the respondents. The mean age was 32.0 ± 7.4 years, the age range was 17 - 49 years, and respondents were majorly in the 30-39 age.

**Table 1:** Socio-demographic characteristics of respondents

Characteristics	Frequency	Percentage
**Age in years**		
10-19	13	2.7
20-29	181'	37.2
30-39	221	45.4
40-49	72	14.8
		
**Religion**		
Christianity	389	79.9
Islam	98	20.1
		
**Marital status**		
Married	409	84.0
Single	69	14.2
Widowed	4	0.8
Separated	5	1.0
		
**Educational status**		
None	12	2.5
Primary	22	4.5
Secondary	139	28.5
Tertiary	314	64.5
		
**Occupation**		
Non-skilled	111	22.8
Semi-skilled	122	25.1
Skilled	213	52.2

Of all respondents, 84.0% were married, 14.2% single, 1.0% separated and 0.8% were widowed. Most of the respondents (79.9%) were Christians while 20.1% were Muslims.

Most of the respondents (64.5%) had tertiary education while only 2.5% had no formal education. The majority of the respondents (52.2%) were engaged in skilled occupation.

Three hundred and seventy six (77.2%) of the respondents had heard of RTI's while 111 (22.8%) of them had not heard of RTI's. Sources of information were: health professionals-127(33.8%), friends and relatives-106(28.2%), print media-45(12%), religious groups-42(11.2%), electronic media-32(8.5%) and school-14(3.7%). Table 2 shows the response of the participants to the modes of acquiring RTI's and knowledge of symptoms. Toilet was the most perceived mode of contracting RTI's (44.6%), followed by sexual intercourse (44.1%) and poor hygiene (24.8%).

**Table 2:** Response to modes of acquiring RTI's and names of RTI's

Characteristics	Frequency	Percentage
**Modes of acquiring RTI's**		
Sexual intercourse	215	44.1
Poor hygiene	121	24.8
Toilet	217	44.6
Unsafe abortion	99	20.3
Unsafe delivery	84	17.2
Genital tract procedures	69	14.2
		
**Names of RTI's**		
Syphylis	78	16.0
Gonorrhoea	114	23.4
HIV/AIDS	36	7.4
Staphylococcus	41	8.4
Candida	48	9.9
Hepatitis	1	0.2
Trichomonas	7	1.4

Gonorrhoea was the commonest RTI named (23.4%) followed by syphilis (16.0%) and candida (9.9%).

Table 3 shows the knowledge of the symptoms and complications of RTI's. Of all respondents, 281 (57.7%) chose vaginal discharge as a symptom of RTI's, 263 (54.0%) chose vaginal itching and 158 (32.4%) chose lower abdominal pain.

**Table 3:** Knowledge of symptoms and complications of RTI's.

Characteristics	Frequency	Percentage
**Knowledge of symptoms**		
Vaginal discharge	281	57.7
Vulval itching	263	54.0
Lower abdominal pain	158	32.4
Painful urination	138	28.3
Genital sore	109	22.4
Pain during menses	101	20.7
Genital/groin swelling	78	16.0
Painful intercourse	75	15.4
		
*Overall knowledge of symptoms		
Poor	273	56.0
Fair	128	26.3
Good	86	17.7
		
**Knowledge of complications**		
Infertility	280	57.5
Cervical cancer	89	18.3
Heavy menses	84	17.2
Ectopic pregnancy	78	16.0
Chronic pelvic pain	78	16.0
Miscarriage	76	15.6
Still birth	71	14.6
Congenital anomaly	58	11.9
		
*Overall knowledge of complications		
Poor	345	70.8
Fair	108	22.2
Good	34	7.0

*A total of 8 effects were scored. Knowledge of 0-2 = poor,

Knowledge of 3-4 = fair, Knowledge of 5 and above = good.

Majority of the respondents knew inability to get pregnant as a complication of RTI's, as indicated by 57.5% of them. Other complications were less known.

Overall knowledge of the respondents about the symptoms of RTI's was poor with only 17.7% having good knowledge and 56.0% demonstrating poor knowledge Overall knowledge of complications was even worse with only 7.0% having good knowledge and 70.8% having poor knowledge.

Table 4 shows that the understandings of the respondents about the effects of contraception on RTI's is poor. 50.5% did not know the effect of condom and 2.5% wrongly believe that it increased the risk of contacting RTIs. Their understandings about the oral contraceptive pills, IUCD and implants with regards to RTI's were also poor.

**Table 4:** Understandings of the effects of contraception on transmission of RTI's

Type of contraception	Frequency	Percentage
**Condom**		
Increase	12	2.5
Decrease	196	40.2
No effect	33	6.8
I don't know	246	50.5
		
**IUCD**		
Increase	22	4.5
Decrease	31	6.4
No effect	56	11.5
I don't know	378	77.6
		
**OCP**		
Increase	11	2.3
Decrease	32	6.6
No effect	54	11.1
I don't Know	390	80.1
		
**Implants**		
Increase	10	2.1
Decrease	13	2.7
No effect	53	10.9
I don't know	411	84.4

One hundred and eighty two of the respondents (37.4%) had experienced symptoms of RTI's in the preceding six months before the study.

The commonest symptom experienced was vaginal discharge which was reported by 106 (21.8%) of the respondents. Other symptoms reported include: vulval itching (17.7%), lower abdominal pain (15.0%), pain with menstruation (8.8%), pain during intercourse (2.7%), genital sore (1.2%) and genital/groin swelling (1.0%).

Table 5 shows that most patients (87.9%) sought medical care when they experienced symptoms of RTI's. Only 9.9% treated self while 2.2% ignored symptoms. Government health centres were the most visited health facility for treatment of RTI's.

**Table 5:** Care seeking behaviour

Characteristics	Frequency	Percentage
**Behaviour with symptoms(n = 182)**		
Ignored symptoms	4	2.2
Treated self	18	9.9
Sought medical care	160	87.9
		
**Care providers in those who sought Medical care(n = 160)**		
Government Health Centre	56	31.5
General Hospital	23	12.9
Private Hospital	34	19.1
Pharmacy	40	22.5
Herbal home	14	7.8
Nursing home	11	6.2
		
**Response to reasons for not seeking medical care (n = 22)**		
Absence of perceived morbidity	2	9.1
Mildness of symptoms	5	22.7
Short duration of symptoms	12	54.5
Financial reasons	3	13.6

15 of the 22 respondents (68.2%) who did not seek medical care gave short duration of symptoms as the reason for their behaviour.

## Discussion

Reproductive Tract Infections account for a major burden of disease globally especially in the developing countries where it is hyperendemic [1]. This is not unconnected with the underlining cultural, traditional and socio- economic factors found in these countries.

Most of the women in the study were in the 30-39 years age group, which constitutes a substantial part of the active reproductive age group and are thus prone to RTI's.

Most of the respondents (77.2%) had heard about RTI's. This is comparable to a similar study conducted in Kenya which showed that over 96% of respondents were aware of RTI's [17]. A study in rural Bangladesh however reported that only 12% of the study population had basic understanding of RTI's [18]. In rural west Bengal 57% had knowledge of RTI's [19].

The great disparity in the proportion of the various populations who were aware of RTIs could be explained from the rural nature of West Bengal and Bangladesh where most of the respondents were illiterates, when compared to the urban settings of Nigeria and Kenya where the literacy level is higher.

In this study, more of the respondents (44.6%) chose toilet as the mode of acquiring RTI's, thereby erroneously attributing more significance to toilet rather than sexual intercourse which was chosen in 44.1% of the respondents as a mode of contracting RTI's. Procedures in the genital tract including abortion were only perceived as a mode of acquiring RTI's in only a few of the respondents. This is in a population where induced abortion is still a major public health problem accounting for about 20,000 deaths annually [20].

In Nigeria, abortion has not been legalised and as such abortions are performed under clandestine conditions where asepsis is not recognised. Most deaths from induced abortion are due to infection [21].

In a Karachi study no respondent mentioned sexual intercourse as a mode of contacting RTI's [22], whereas in Lima, Peru, knowledge of sexual contact as one way of transmitting RTIs was high [23].

This study shows that knowledge of the names of RTI's was poor as only 23.4% mentioned gonorrhoea as a cause of RTI's while 16.0% and 9.9% mentioned syphilis and candida respectively.

Vaginal discharge was the most perceived symptom of RTI's. This was not unexpected as previous reports from Nigeria have shown vaginal discharge as a common complaint in self referred cases to the gynaecological clinic [24,25]. It was also not surprising that pruritus vulvae was the second commonest perceived symptom of RTI's as *Trichomonas vaginalis *and *Candida albicans *which are common causes of vaginal discharge also cause pruritus.

It is worthy of note that genital sore was poorly perceived as a symptom and because of its association with increased incidence of HIV/AIDS, there is need to make the populace aware of the importance of the symptom as it relates to HIV transmission.

Inability to get pregnant topped the list of reported complications of RTI's. This is perhaps a reflection of the importance attached to childbearing in our society whereby any affectation of the genital region may be perceived as preventing pregnancy. Unfortunately, ectopic pregnancy with its enormous contribution to maternal morbidity and mortality in our country was poorly perceived as a complication of RTI's. The perceptions of other complications such as cancer of the cervix as related to RTIs were also poor.

The overall knowledge of symptoms and complications in this study were poor. There was poor knowledge of contraception and its role in preventing RTI's. This is however not surprising as the contraceptive prevalence in Nigeria is well below 10% [26]. The major obstacles to deriving benefits from contraception among our women include ambivalence towards modern contraception which is particularly grounded in the fear of side effects, poor quality services, and opposition from family members or influential members of the community [26].

Regarding prevalence of symptoms, 37.4% of respondents had experienced at least one symptom in the previous 6 months. This finding is similar to 41.3% reported from Shimla City, Northern India [27], but much lower than that reported from a study in west Bengal, India, where 66.1% of women of reproductive age reported symptoms related to the reproductive tract [19].

Vaginal discharge was the commonest symptom experienced in this study in contrast to a study in rural Lebanon [28] where lower abdominal pain (41.1%), vaginal itching (38.5%) and painful coitus (40.7%) were the leading symptoms experienced. Only 24.5% had experienced vaginal discharge.

In this study, the majority of the respondents (87.9%) sought medical care. This may be due to the fact that infertility is known by many to result from RTI's and to prevent such dreaded complications women tend to seek medical care rather than face the consequences. It also indicates that intervention to reduce morbidity arising from RTIs should be directed mainly towards preventing RTI's rather than cure as most of the respondents will seek treatment when symptomatic.

Government Health Centre's were the main facilities where treatment was sought. This may be largely due to the fact that many health centre's are located in each local government area close to the people. Their services are also very cheap and sometimes free. In addition they are not as busy as the general hospitals which serve as a referral centre for more complicated cases and patients have to wait for long hours before being attended to.

This finding is different from what is reported in West Bengal where most respondents (41.5%) patronized private doctors [19]. Perhaps the expensive nature of private hospitals in Lagos could explain this difference.

Another interesting consideration in the choice of care providers is the unrestricted access of the populace to medications, including antibiotics without prior medical consultation. Pharmaceuticals are obtained over the counter and often self administered on the basis of prior personal experience or lay advice from neighbours and family members. This results in a lot of patients patronizing pharmacies even more than they patronize the general hospitals or private doctors as shown in this study.

It is important to understand the limitations of our study. For prevalence of symptoms and care seeking behaviour of the participants, we relied upon self reported answers to events that may have occurred earlier and these may be subject to recall and reporting bias. Also, severe symptoms are likely to be remembered longer than mild symptoms.

## Conclusion

The result of this study shows that even though most of the respondents had heard of RTI"s, they demonstrated poor understanding of the subject.

The prevalence of symptoms of RTI's in this study is high compared to that reported in other studies and most of those who reported symptoms sought medical care.

This implies that health intervention measures directed towards reducing morbidities from RTI's need not focus mainly on treatment of RTI's but rather on disease preventing strategies. These include educating women about ways of preventing RTI's such as the avoidance of high risk sexual behaviours and use of barrier contraceptive methods as reproductive tract infections are often assymptomatic.

RTI preventive programmes should be integrated into other reproductive health care programmes such as family planning, maternal and child health services with a view to providing a broad based reproductive health care.

## Competing interests

The authors declare that they have no competing interests.

## Authors' contributions

KAR conceived the study and participated in its design and co-ordinated the training of interviewers, data collection, analysis and wrote the first draft of the paper. AAA participated in the study design, data collection, analysis and helped to draft the manuscript. FMA participated in the study design, data collection, analysis and helped to draft the manuscript. OIA participated in the study design, analysis and helped to draft the manuscript. All authors read and approved the final manuscript.

## Pre-publication history

The pre-publication history for this paper can be accessed here:

http://www.biomedcentral.com/1472-6874/10/8/prepub
